# Book Reviews in Brief

**Published:** 1914-05-09

**Authors:** 


### Book Reviews in Brief.

Dr. Chavasse's Advice to a Mother. Seventeenth Edition. Revised by T. D. Lister, M.D. (London: J. and A. Churchill. 1913. Price 1s. 6d. net.)

Invaluable as the two famous pioneer books containing Dr. Chavasse's advice, to a wife and a mother respectively, have proved to be, the earlier editions naturally could not reflect modern practice and precept. It is, however, satisfactory to know that they have been brought thoroughly up to date by experts; and this new issue of the book for mothers has been so radically revised and recast by Dr. Lister that we almost doubt whether the original edition would bear any resemblance to its offspring. The whole revision has been judicious, and very considerable improvements have been effected by the editor. The present (seventeenth) edition completes the 340th thousand, a fine testimony to the popularity of "Chavasse." We see no reason why in course of time the circulation should not reach a million, for it is in the front rank of books of its class, and the demand for it is only likely to cease when the birth-rate drops completely to zero.

Diseases of the Skin. By David Walsh, M.D. (London: Baillière, Tindall and Cox. 1913. Pp. 298. Price 6s. net.)

The extension of scientific knowledge about diseases of the skin has been very rapid during the last few years, and modern text-books in endeavouring to summarise all researches and to be up to date at all costs frequently contain much that is of little practical service to the medical student and the general practitioner. This is a pitfall which Dr. Walsh avoids. He is careful to describe the latest advances when and where they are indispensable to a proper knowledge of modern practice; but he eschews the inessential skilfully, and so simplifies his book and makes it much easier reading for those to whom it is addressed. For instance, in discussing the treatment of ringworm, he mentions his views of the successes and of the limitations of treatment by *x*-rays; but he does not enter into any details whatever of their administration, because the matter is so technical that it is best left to specialists. Some of Dr. Walsh's views, which are always expressed firmly and tersely, may not be strictly orthodox, but we should be sorry to say that they are wrong. There is a bad misprint in a prescription on p. 137, but otherwise the "get-up" of the book reflects as much credit on the publisher as its contents do upon the author.

A Short Practice of Midwifery for Nurses. By Henry Jellett, M.D. Fourth Edition, Revised. (London: J. and A. Churchill. (Dated) 1914. Pp. 508. Price 7s. 6d. net.)

This text-book, now in its fourth edition, bears a strong resemblance to the similar book by the same author designed for medical practitioners. In general terms it possesses the merits of the larger work, which are great, and also its weak points, which are few. The process of adaptation of the medical text-book for a different circle of readers has, if anything, not been quite as complete as some authors and teachers might think necessary; and, indeed, Dr. Jellett frankly admits that there is much in this book that concerns nurses very indirectly. The ambitious woman, however, who aims rather at being an expert midwife than a maternity nurse (and especially she who has had a "general training" to begin with), will find every section of this book useful and valuable; and its popularity, as shown by the demand for successive editions, is based upon a foundation of real worth and utility.

